# Employing Tribe to Study RNA Interactions of Ataxin-2 in *Drosophila* S2 Cells

**DOI:** 10.21769/BioProtoc.5613

**Published:** 2026-03-05

**Authors:** Shiva Kumar, Omkar Koppaka, Khushboo Agrawal, Baskar Bakthavachalu

**Affiliations:** 1 School of Biosciences and Bioengineering, Indian Institute of Technology Mandi, Mandi, Himachal Pradesh, India; 2 Tata Institute for Genetics and Society, UAS-GKVK Campus, Bellary Road, Bangalore, Karnataka, India

**Keywords:** Ataxin-2, *Drosophila*, TRIBE, S2 cells, RNA binding proteins

## Abstract

RNA-binding protein (RBP)–RNA interactions are fundamental for gene regulation and cellular homeostasis. Ataxin-2 is an RBP that has been shown to play an instrumental role in pathophysiological processes by binding to mRNA. Methods such as RNA immunoprecipitation (RIP), cross-linking immunoprecipitation (CLIP), and their variants can be used to study the interactions between Ataxin-2 and its targets, although their high sample requirements and labor-intensive workflows can limit their widespread use. RNA editing-based approaches, such as targets of RBPs identified by editing (TRIBE), provide effective alternatives. TRIBE enables transcriptome-wide identification of RBP targets by inducing site-specific adenosine-to-inosine (A-to-I) editing, which is subsequently detected through high-throughput RNA sequencing in both in vivo and in vitro systems. Compared to in vivo models, cell lines offer a rapid and flexible experimental design. *Drosophila* S2 cells are a commonly used insect cell line to investigate RNA–protein dynamics and serve as a versatile platform for studying RBP function. Here, we describe a protocol used for identifying RNA targets of Ataxin-2, a versatile RBP involved in post-transcriptional and translational regulation, in S2 cells using TRIBE. This method allows rapid, efficient, and reliable identification of Ataxin-2-associated RNA targets and can be readily applied to other RBPs.

Key features

• Streamlined workflow for identifying RNA targets of RBPs using TRIBE in *Drosophila* S2 cells.

• TRIBE highlights the sites and nature of interactions between RBPs and RNA.

• TRIBE overcomes the limitations of conventional RBP–RNA interaction studies like RIP, CLIP, and their variants.

## Graphical overview


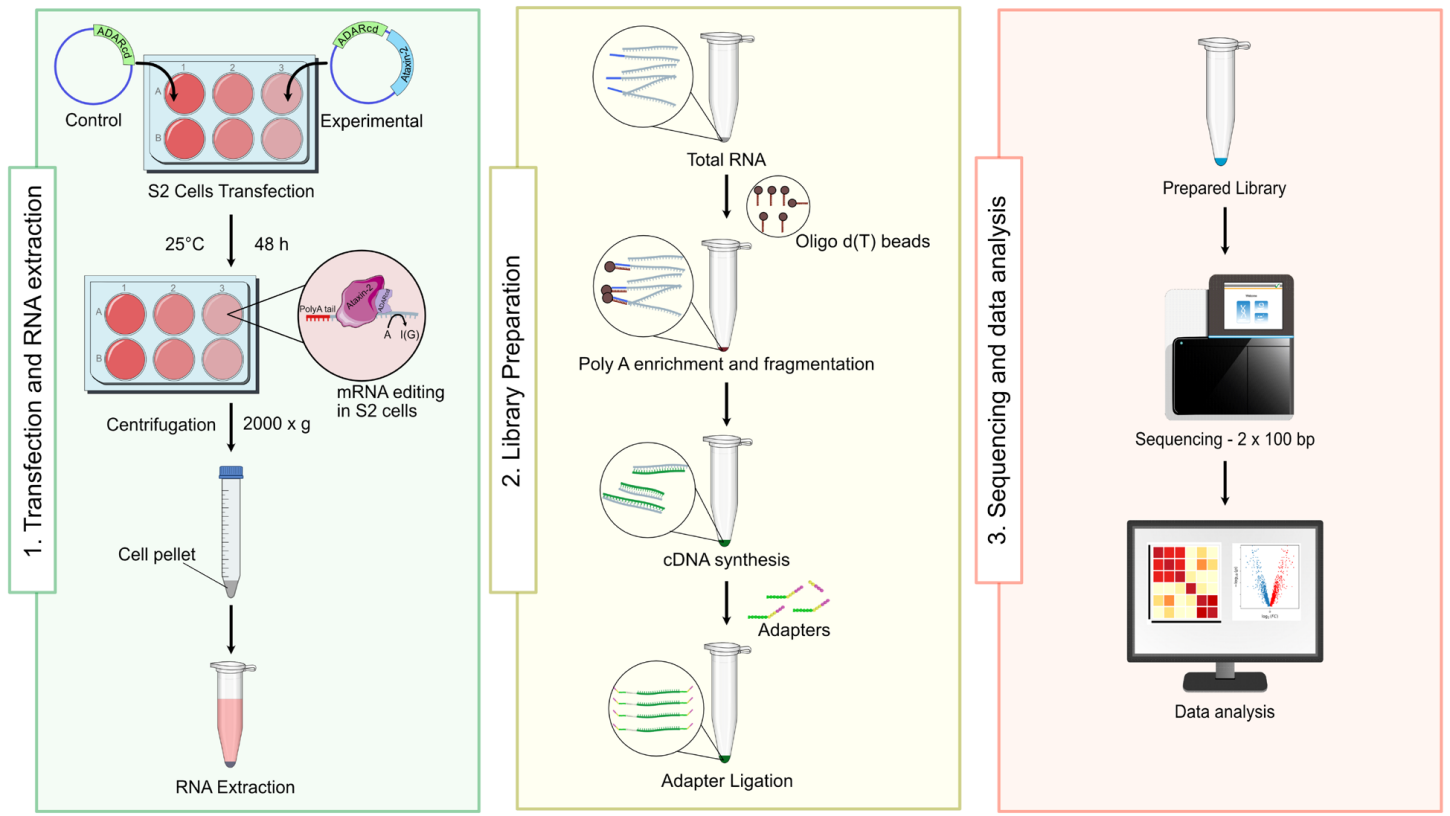


## Background

Gene regulation is fundamental for maintaining cellular homeostasis and optimal physiological function. A key component of this regulation involves interactions between RNA-binding proteins (RBPs) and their RNA targets [1]. One such RBP, Ataxin-2, is evolutionarily conserved across organisms ranging from yeast to humans, where it plays critical roles in RNA metabolism, including regulation of mRNA stability and translation. Beyond its functions in RNA regulation, Ataxin-2 is involved in broader cellular processes such as calcium signaling and maintenance of circadian rhythms. Dysregulation of Ataxin-2 has been implicated in a range of pathological conditions, including neurodegenerative diseases, metabolic disorders, and various cancers [2]. Due to its regulatory roles in pathophysiological processes mediated through interactions with its RNA targets, studying these interactions is essential for addressing fundamental questions regarding the function of Ataxin-2 in these processes. To this end, numerous experimental approaches have been developed to characterize RNA-binding proteins and RNA interactions [3]. Immunoprecipitation-based methods such as RNA immunoprecipitation (RIP), crosslinking immunoprecipitation (CLIP), and their variants are widely used [4]. However, they have several drawbacks, including labor-intensive workflows, a large quantity of samples, dependence on high-quality antibodies, and the risk of capturing nonspecific interactions [5,6].

More recently, RNA editing–based techniques such as TRIBE (Targets of RNA-Binding Proteins Identified By Editing) and STAMP (Surveying Targets by APOBEC-Mediated Profiling) have gained prominence due to their relative simplicity and minimal sample requirement [7,8]. TRIBE leverages the activity of adenosine deaminase acting on RNA (ADAR), an RNA-editing enzyme that catalyzes the deamination of adenosine (A) to inosine (I) within double-stranded RNA regions. TRIBE involves fusion of the catalytic domain of ADAR (ADARcd) to the RBP of interest to identify RBP–RNA interactions by detection of A-to-I edits on target RNAs using high-throughput RNA sequencing [8].

RBP–RNA interaction studies can be performed in cultured cell lines due to their ease of manipulation, rapid experimental setup, and straightforward analysis. While cell lines have limitations, including heterogeneous gene expression patterns and inability to fully recapitulate cell type–specific transcriptional programs, they remain powerful tools for investigating RNA–protein interactions and studying their general RNA regulation mechanisms. TRIBE and its enhanced variant, HyperTRIBE, have both been successfully applied in animal cell lines, plants, and yeast [8–12].

In *Drosophila* research, S2 cells are the preferred cell lines. In this protocol, adapted from Petrauskas et al. and Singh et al. [13,14], we describe a detailed workflow for performing TRIBE in *Drosophila* S2 cells. This method enables reliable detection of Ataxin-2 targets through ADAR-mediated RNA editing and provides a robust framework for studying RNA–RBP interactions with high accuracy and reproducibility.

## Materials and reagents

**Cell lines and culture reagents**

1. Cell Lines: *Drosophila* S2R+ cells (DGRC, Indiana University)

2. Growth media and supplements:

a. Schneider's insect medium (Himedia, catalog number: IML003A)

b. Fetal bovine serum (FBS) (Himedia, catalog number: RM9955)

c. Penicillin and streptomycin (Himedia, catalog number: A001A)

**Reagents and kits**

1. Plasmids

a. pJFRC7-20XUAS-IVS-8_ADARcd only construct [14]

b. pJFRC7-20XUAS-IVS-8_Ataxin-2-ADARcd construct [14]

2. Transfection

a. TransIT-X2 (MirusBio, catalog number: MIR 6000)

3. RNA Extraction, library preparation, and sequencing

a. TRIzol reagent (Invitrogen, catalog number: 15596026)

b. 1-Bromo-3-chloropropane (Thermo Fisher Scientific, catalog number: AC106860501)

d. c. Isopropanol (Merck, catalog number: I9516-500ml)

e. Ethanol (Honeywell, catalog number: 32221-500ml)

f. NEBNext poly(A) mRNA magnetic isolation module (New England Biolabs, catalog number: E7490L)

g. NEBNext Ultra II Directional RNA Library Prep kit (New England Biolabs, catalog number: E7765L)

h. Unique Dual Index UMI Adaptors RNA (New England Biolabs, catalog number: E7416L)

i. NovaSeq 6000 SP Reagent kit v1.5 (Illumina Inc., catalog number: 20040719)

## Equipment

1. Incubator set at 25 °C (PHCbi, model: MIR-154)

2. Cooling centrifuge (Neuation, catalog number: M24PR-211)

3. Vortex (Neuation, catalog number: VM-2110)

4. Thermal cycler (Applied Biosystems, catalog number: 4484073)

5. Qubit fluorometer (Thermo Fisher Scientific, catalog number: Q33327)

6. Agilent TapeStation (Agilent, catalog number: G2991BA)

7. Magnetic rack (New England Biolabs, catalog number: S1515S)

8. Illumina Sequencing platform NovaSeq 6000 (Illumina Inc., catalog number: 20012850)

9. PC Workstation (preferred, but analysis can be done on HPC or laptop as well)

## Software and datasets

**Table:** 

Tool	Source	Purpose
FastQC	https://www.bioinformatics.babraham.ac.uk/projects/fastqc/	For performing a quality check of the FASTQ files.
Trimmomatic	https://github.com/usadellab/Trimmomatic	Removes the adapter and other low-quality sequences from the FASTQ file.
STAR	https://bioinformaticshome.com/tools/rna-seq/descriptions/STAR.html	Aligns the FASTQ files to a reference genome.
Samtools	https://www.htslib.org/	Perform operations on the SAM files obtained from alignment.
Picard-tools	https://broadinstitute.github.io/picard/	Removes PCR duplicates that may result in false positives.
HyperTRIBE software	https://github.com/rosbashlab/HyperTR	-
trim_and_align.sh	-	This script is used for trimming to remove low-quality and adapter sequences, followed by alignment to a reference genome (*Drosophila melanogaster* dm6 reference genome in our case). The output file generated as a SAM file is then sorted with respect to coordinates, and duplicate reads are removed.
load_table.sh	-	Take the SAM file produced in the previous step (containing all the mapped reads with A to G editing information) and convert it into a matrix-style table that is compatible with an SQL database, which allows for easier and structured data handling.
rnaedit_wtRNA_RNA.sh	-	The rnaedit_wtRNA_RNA.sh script compares experimental and control RNA samples against wildtype (non-edited) RNA, allowing detection of editing events above the natural RNA editing background.
MySQL or MariaDB	https://mariadb.org	Used for the conversion of the unstructured SAM files to an easier-to-handle structured database.
R and RStudio	https://www.r-project.org/ https://rstudio-education.github.io/hopr/starting.html	For downstream applications on the raw edit files generated from the control and experimental samples.

*Notes:*

*1. All software used for data analysis is open-source/freely available (provided in the hyperlinks on the above table).*

*2. We recommend including at least two replicates for each condition when performing TRIBE to improve reproducibility. Further paired-end sequencing yields better read depth, which further enhances target identification.*

*3. We also advise generating a minimum of 15 million reads per sample for* Drosophila *and 25–30 million reads for vertebrates. This depth typically provides sufficient coverage to detect predominant RNA targets.*

## Procedure

**A. S2R+ cell culture and transfection**

1. Culture 1 × 10^6^ S2R+ cells/well in a 6-well plate overnight at 25 °C in 2 mL of Schneider's insect medium containing 10% FBS and 0.5% penicillin and streptomycin.

2. Bring TransIT-X2 to room temperature and vortex gently.

3. Meanwhile, dilute 2.5 μg of plasmid DNA in 250 μL of serum-free medium in a sterile tube and gently tap mix.

4. Add 7.5 μL of TransIT-X2 to the diluted DNA mixture and gently tap the tube to mix the contents. Incubate at room temperature for 30 min.

5. Add TransIT-X2 + DNA complex mixture dropwise to the wells containing growing S2R+ cells.

6. Gently rock the culture plate to evenly distribute the complexes and incubate at 25 °C for 48 h.

*Note: To improve reproducibility among replicates, the constructs can be co-transfected with a GFP plasmid at a 9:1 ratio or expressed as RBP-ADARcd fused with GFP separated by T2A translation skip sequence. Combining this with FACS facilitates the selection of transfected cells, thereby improving the reproducibility and efficiency of this method.*

**B. RNA extraction**

1. Collect 1 × 10^6^ transfected S2R+ cells in a centrifuge tube and spin at 2,000× *g* for 10 min to pellet the cells. Add 1 mL of TRIzol reagent to the cell pellet and mix well by inverting the tube 5–6 times gently.

2. Add 100 μL of 1-Bromo-3-chloropropane and mix briefly for 10–15 s. Incubate for 10 min at room temperature.

3. Centrifuge at 12,000× *g* for 15 min at 4 °C. The mixture separates into a lower organic phase, an interphase, and a colorless upper aqueous phase.

4. Carefully transfer the upper aqueous phase to a new tube without disturbing the interphase.

5. Add 0.5 mL of 100% isopropanol to the aqueous phase and incubate for 10 min at room temperature.

6. Centrifuge the tube at 12,000× *g* for 10 min at 4 °C and discard the supernatant without disturbing the RNA pellet.

7. Wash the RNA pellet with 1 mL of freshly prepared 80% ethanol.

8. Centrifuge at 12,000× *g* for 10 min at 4 °C, remove ethanol, and air dry the RNA pellet.

9. Resuspend the pellet in 20–50 μL of RNase-free water and quantify the RNA using a fluorometer (Qubit).

10. Check the RNA quality using Agilent TapeStation.

**C. PolyA RNA enrichment and RNA fragmentation**

*Note: The PolyA enrichment and RNA fragmentation were performed as described in the manufacturer’s protocol of NEBNext Ultra II Directional RNA Library Prep kit. The steps are described below*:

1. In a fresh tube, mix 20 μL of NEBNext oligo d(T)_25_ beads and 100 μL of RNA binding buffer (2×) and mix by pipetting.

2. Place the tube on a magnetic rack at room temperature for 2 min or until the solution becomes clear. Discard the supernatant from the tube without disturbing the beads.

3. Remove the tube from the magnetic rack and add 100 μL of NEBNext RNA binding buffer (2×) per reaction to the beads and mix them to homogenize.

4. Place the tubes on the magnetic rack and incubate. Once the solution becomes clear, discard the supernatant.

5. Add 50 μL of NEBNext RNA binding buffer (2×) to the beads and mix by pipetting until homogeneous.

6. In another fresh tube, dilute 1 μg of total RNA with nuclease-free water to a final volume of 50 μL in a PCR tube.

7. Add 50 μL beads (from step C5) to the RNA sample tube. Mix thoroughly by pipetting.

8. Place the tube in a thermal cycler and incubate at 65 °C for 5 min to denature the mRNA and facilitate its binding to the oligo d(T)_25_ beads.

9. Remove tubes from the thermal cycler and incubate at room temperature for 5 min. Then, place it on the magnetic rack until the solution becomes clear.

10. Discard the supernatant without disturbing the beads; do not remove the tubes from the magnetic rack.

11. Wash the beads by gently adding 200 μL of NEBNext wash buffer to the tubes to remove the unbound RNA and mix by gentle pipetting.

12. Place the tube on a magnetic rack at room temperature until the solution becomes clear. Discard the supernatant and remove the tube from the magnetic rack.

13. Repeat steps C11–12 two times.

14. Add 50 μL of Tris buffer (provided in the kit) and gently mix.

15. Place the tube on a thermal cycler set to 80 °C for 2 min and then cool to 25 °C.

16. Add 50 μL of RNA binding buffer (2×) to the sample to allow the mRNA to re-bind to the beads, mix by pipetting, and incubate the tube at room temperature for 5 min.

17. Place the tube on a magnetic rack until the solution becomes clear.

18. Discard the supernatant while taking care not to disturb the beads.

19. Wash the beads by adding 200 μL of wash buffer (provided in the kit). Place the tube on the magnetic rack until the solution becomes clear and discard the supernatant.

20. Remove the tube from the magnetic rack.

21. Prepare a reaction mixture with 8 μL of first-strand synthesis reaction buffer and 2 μL of random primer. Make up the volume to 20 μL with nuclease-free water in a fresh tube.

22. To elute the mRNA from the beads and fragment them, add 11.5 μL of the mix prepared in step C21 to the mRNA and pipette up and down 6 times to resuspend the beads.

23. Incubate the sample in a thermal cycler at 94 °C and hold at 4 °C.

24. Immediately transfer the tube to ice for 1 min as soon as it is cool enough to handle (~65 °C).

25. Quickly spin down the tube in a microcentrifuge to collect the liquid from the sides of the tube and place it on the magnetic rack until the solution is clear.

26. Collect the fragmented mRNA by transferring 10 μL of the supernatant to a nuclease-free 0.2 mL PCR tube and place on ice. Proceed directly to cDNA first-strand synthesis.

**D. cDNA synthesis**

1. For cDNA first-strand synthesis, prepare the reaction mixture on ice by adding 10 μL of fragmented and primed RNA mixture (from step C26), 8 μL of NEBNext strand specificity reagent, and 2 μL of NEBNext first-strand synthesis enzyme mix, and mix thoroughly by pipetting up and down at least 10 times.

2. Incubate the sample in a thermal cycler with the following program: 25 °C for 10 min, 42 °C for 15 min, 70 °C for 15 min, and hold at 4 °C.

3. For cDNA second-strand synthesis, prepare a mixture of 20 μL of first-strand synthesis product (from step D2), 8 μL of NEBNext second-strand synthesis reaction buffer with dUTP mix, and 4 μL of NEBNext second-strand synthesis enzyme mix, and make up the volume to 80 μL with nuclease-free water. Mix well while on ice and incubate in a thermal cycler for 1 h at 16 °C.

4. Purify the double-stranded cDNA by adding and mixing 144 μL (1.8×) of resuspended SPRIselect beads to the second-strand synthesis reaction (~80 μL) (from step D3).

5. Incubate for 5 min at room temperature and place the tube on a magnetic rack until the solution is clear. Discard the supernatant without disturbing the beads.

6. Add freshly prepared 80% ethanol, incubate for 30 s, and then discard the supernatant. Repeat this step twice.

7. Place the tube on a magnetic rack and air-dry the beads.

8. Remove the tube from the magnet. Add 53 μL of 0.1× TE buffer and mix well. Incubate at room temperature for 2 min and place on a magnetic rack.

9. Transfer the supernatant to a clean PCR tube.

10. For end prep of cDNA library, prepare a mixture of 50 μL of second-strand cDNA synthesis product (from step D9), 7 μL of NEBNext Ultra II end prep reaction buffer, and 3 μL of NEBNext Ultra II end prep enzyme mix, and mix them well.

11. Incubate the mixture in a thermal cycler with the following program: 20 °C for 30 min, 65 °C for 30 min, and hold at 4 °C.

**E. Adaptor ligation using indexed-UMI adaptor**

1. Dilute the thawed NEBNext unique dual index UMI RNA adaptor on ice with ice-cold UMI adaptor dilution buffer (Table 1) and briefly spin if needed.

**Table 1.** Recommended dilution of NEBNext unique dual index UMI RNA adaptor.

Total RNA input	Dilution requirement
1,000–250 ng	No dilution
249–100 ng	10-fold dilution in UMI adaptor dilution buffer
99–10 ng	50-fold dilution in UMI adaptor dilution buffer

*Note: Keep the adapters on ice at all times.*

2. Prepare the ligation reaction mixture on ice with 60 μL of end prep DNA (from step D11), 5 μL of diluted adaptors, 1 μL of NEBNext ligation enhancer, and 30 μL of NEBNext Ultra II ligation master mix, and pipette mix. Incubate at 20 °C for 15 min in a thermal cycler.

3. Add 3 μL of USER enzyme to the ligation reaction mixture from (from step E2) and mix well by pipetting. Incubate in a thermal cycler at 37 °C for 15 min.

4. Proceed immediately with the purification of the ligation reaction product by adding 70 μL (0.7×) of resuspended SPRIselect beads. Incubate for 10 min at room temperature after mixing well on a vortex.

5. Put the tube on the magnetic rack until the solution becomes clear and discard the supernatant.

6. Wash with freshly prepared 200 μL of 80% ethanol while on the magnetic rack. Repeat this step twice.

7. Completely remove the ethanol and air-dry.

8. Remove the tube from the magnetic rack and add 22 μL of 0.1× TE buffer to the beads. Mix well with pipetting.

9. Incubate for 2 min at room temperature and put on the magnetic rack until the solution becomes clear.

10. Transfer 20 μL of the supernatant to a clean PCR tube without disturbing the beads.

11. For PCR enrichment of the adaptor-ligated DNA, prepare the PCR reaction with 20 μL of UMI adaptor-ligated DNA (from step E10), 25 μL of NEBNext Ultra II Q5 master mix, and 5 μL of NEBNext primer mix. Mix well.

12. Place the tube on a thermal cycler and run the program described in Table 2.

**Table 2.** PCR program for adaptor-ligated DNA enrichment.

Cycle step	Temperature (°C)	Time	Cycles
Initial denaturation	98	30 s	1
Denaturation	98	10 s	8–17
Annealing/extension	65	75 s	
Final extension	65	5 min	1
Hold	4	∞	

13. For purification of the PCR product, add 45 μL (0.9×) of resuspended SPRIselect beads to the PCR product mixture and mix well by pipetting.

14. Incubate for 5 min at room temperature and put the tube on a magnetic rack until the solution is clear.

15. Discard the supernatant without disturbing the beads.

16. Add 200 μL of freshly prepared 80% ethanol to the tube while on the magnetic rack. Incubate at room temperature for 30 s and then discard the supernatant. Repeat this step twice.

17. Air-dry the beads on a magnetic rack.

18. Remove the tube from the magnetic rack and add 23 μL of 0.1× TE buffer to the beads. Mix well by pipetting.

19. Quickly spin the tube in a centrifuge and incubate for 2 min at room temperature. Place the tube in the magnetic rack until the solution is clear.

20. Transfer 20 μL of the supernatant to a clean PCR tube.

21. Check the quality of the prepared library on the Agilent TapeStation.

**F. Sequencing**

1. Load the PCR-enriched adaptor-ligated DNA to the Illumina Sequencer platform NovaSeq 6000 paired-end flow cell and set the parameter to 2× 100 bp with dual indexing for generating paired-end strand-specific data.

2. For sequencing reagent, use NovaSeq 6000 SP Reagent kit and run the setup, priming, and fluidics recommended by the manufacturer.

## Data analysis

**A. Samples required for TRIBE data analysis**

1. Background sample: ADAR is an important enzyme for regulating gene expression as well as for the diversity at the level of proteins and S2 cells. It also possesses endogenous ADAR activity, which can introduce edits into a distinct subset of RNA targets. Therefore, including an untransfected sample allows for subtracting the background arising from both endogenous ADAR-mediated edits and any existing single-nucleotide polymorphisms (SNPs) during downstream analyses.

2. Control sample: Although TRIBE uses only the catalytic domain of ADAR, some off-target editing may still occur. To account for this, an ADARcd-only control sample is included. Edits observed in this control can be subtracted from the experimental sample to ensure specificity.

3. Experimental sample: This sample contains the RNA-binding protein of interest fused to the ADARcd fragment, enabling the identification of RBP-associated RNA targets.

**B. Protocol**

**Figure 1.**
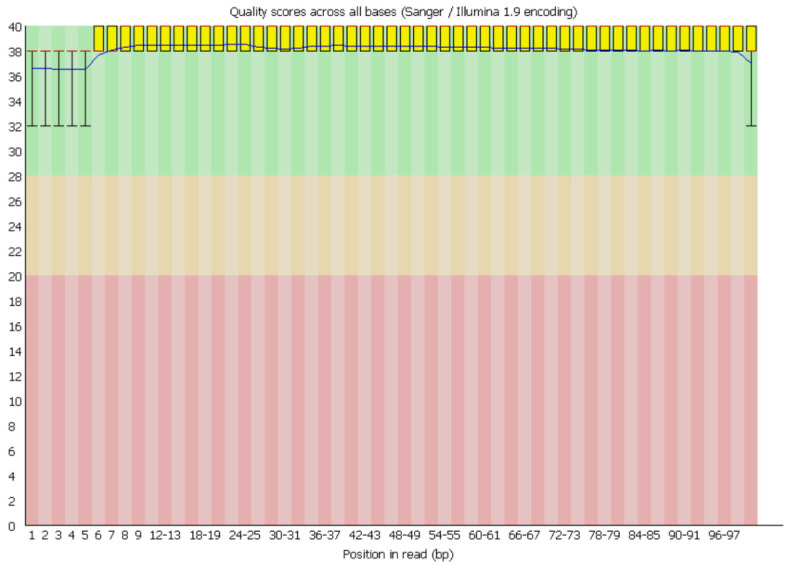
Representative FastQC per-base sequence quality plot for high-quality reads. The x-axis indicates the base position along the sequencing reads, while the y-axis shows the Phred quality score, a measure of base-calling accuracy. Quality scores < 20 are typically considered poor (red zone), scores between 20 and 28 indicate moderate quality (yellow zone), and scores > 28 reflect high-quality bases (green zone). In this dataset, all reads fall within the >28 range, indicating overall good sequencing quality.

1. Obtain the raw FASTQ files generated from sequencing and perform quality control using FastQC.

2. Review the FastQC reports to ensure that the read quality is predominantly in the green zone (Figure 1) before proceeding with downstream analyses.

3. Download the latest reference genome (FASTA) and annotation file (GTF) to build the genome index, which is mandatory for the alignment. For our analysis, we used the *Drosophila melanogaster* dm6 reference genome obtained from iGenomes.

*Note: Use the following command to generate the genome index:*

STAR --runThreadN <No. of threads> --runMode genomeGenerate --genomeDir <index_directory> --genomeFastaFiles <FASTA_file> --sjdbGTFfile <GTF_file>

4. Edit file paths in the trim_and_align.sh script as required. Example:

star_indices="path/to/your/genome/index"

TRIMMOMATIC_JAR="/path/to/your/trimmomatic.jar"

PICARD_JAR="/path/to/picard.jar"

5. Run the script to obtain a sorted SAM file. This SAM file must be converted into a matrix format compatible with an SQL database. Use the load_table.sh script for this conversion.

6. Before running load_table.sh, log into your MySQL or MariaDB server and:

a. Create a new database to store processed data.

b. Record the database name, username, and password for later use.

7. The script uses accessory files such as load_matrix_data.pl (in the HyperTRIBE directory), which must be edited with the database information (from step 6b):

In the load_table.sh script, update the following fields:

my $host = "localhost";

my $database = "dmseq";

my $user = "root”; # MySQL username

my $password = ""; # MySQL password (if any)

*Note: Record all this information for step B12.*

samfile=<Name_of_the_SAM_file>

tablename=<Custom_table_name_for_matrix_data>

expt=<Experiment_identifier>

tp=<Replicate_or_timepoint_identifier>

8. Running the script will convert SAM files into matrix files and upload them to the SQL database for the background, control, and experimental samples.

9. To generate raw edit files for the control and experimental samples (with background edits removed), run the rnaedit_wtRNA_RNA.sh script.

10. Similar to load_table.sh, the wtRNA_RNA.sh script must be directed to the SQL database containing the matrix files.

11. Open find_rnaeditsites.pl and update the database connection fields as described in step B8.

12. Using the parameters recorded in step B9, complete the following fields in rnaedit_wtRNA_RNA.sh:

gDNAtablename=<Table_name_for_background_sample>

gDNAexp=<Experiment_name_for_background_sample>

gDNAtp=<Timepoint_for_background_sample>

RNAtablename=<Table_name_for_control_or_experimental_sample>

RNAexp=<Experiment_name_for_control_or_experimental_sample>

This will generate raw edit files for the control and experimental samples, with background edits subtracted. These files must now be processed to obtain the final list of edits.

13. Apply filtering thresholds within the same script to remove low-confidence edits.

# Create a $1% threshold edit file

edit_threshold= $1

read_threshold= $2

*Note: The read coverage and edit % can be determined by the protein of interest and read depth. In our analysis, we have used an edit percentage of 15%. While a 15% editing threshold increases confidence in target identification, this value could also reduce the number of targets identified, which could potentially lead to false negatives.*

14. Process data using custom RStudio scripts:

a. Use control sample edits to eliminate ADARcd-only off-target edits.

b. Intersect the filtered edits between the experimental replicate 1 and experimental replicate 2.

c. The overlapping edits represent the final list of target sites and their editing positions.

15. Optional downstream analyses. Once the final target list is generated, you may perform additional analyses, such as:

a. Validation of a subset of targets using PCR amplification, followed by Sanger sequencing, as shown in Singh et al. [14] (Figure 1E or Supplementary Figure 1D).

b. Gene ontology (GO) analysis to identify enriched biological processes, cellular components, and molecular functions.

c. Metagene profiling, including editing-density plots across transcript regions (5′UTR, CDS, 3′UTR).

d. Pathway analysis to determine regulatory or signaling pathways associated with target genes.

e. Additional visualizations such as heatmaps, edit-frequency distributions, motif enrichment, or comparisons with published RBP-binding datasets could also be performed.

**Summary**

This protocol has been adopted from [13,14]. TRIBE identifies RNA targets of a specific RNA-binding protein by fusing it to the catalytic domain of the RNA-editing enzyme ADAR. The resulting fusion protein is expressed in defined cell types, where the RBP binds its target RNAs, and the ADAR catalytic domain deaminates nearby adenosines to inosines. Total RNA is then isolated and subjected to high-throughput sequencing. During sequencing, inosines are read as guanosines, enabling detection of editing events. RNA targets are identified through computational filtering for reproducible adenosine-to-guanosine mutations that occur at levels significantly above the genomic background across biological replicates.

Here, we have established an adaptable and reproducible pipeline for mapping the RNA targets of Ataxin-2 in *Drosophila* S2 cells. This protocol details a streamlined workflow while bypassing the limitations of conventional RBP–RNA interaction studies and enables direct capture of Ataxin-2 targets. TRIBE has also been extended to identify the targets of other RBPs, such as Hrp48 in *Drosophila* [8], thereby highlighting it as a versatile toolkit for identifying RBP targets and the significance of their interactions in cellular physiology.

## Validation of protocol

**Figure 2.**
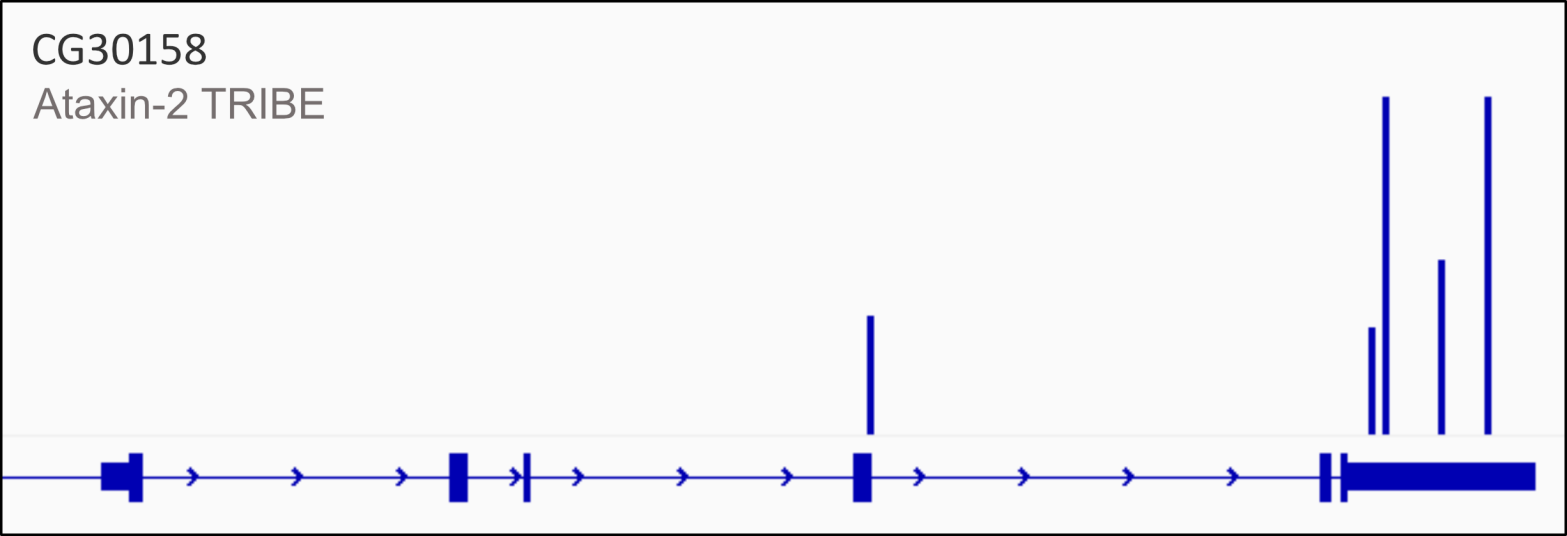
Representative edited gene track for Ataxin-2 targets. CG30158, one of the targets of Ataxin-2 identified by TRIBE analysis, shows editing events within its 3′ UTR (represented by blue bars) in the final edited gene track after removal of background and control-derived edits.

Targets can be validated either through visualization of the gene edit tracks, as represented in Figure 2, or by using Sanger sequencing. This protocol has been used and validated in [14] (Figure 3 and Figure supplements 4, 5, and 6). The cited figures represent the validation of a strong Ataxin-2 target, 14-3-3 epsilon, using Sanger sequencing and mapping the A-to-G modifications to the TRIBE edits. Data has also been made available publicly and can be accessed on Gene Expression Omnibus (GEO): GSE196739.

## General notes and troubleshooting

1. RNA integrity number (RIN) is a critical parameter for getting good-quality data. Here, we recommend RIN > 7.

2. While RBPs are ubiquitously expressed, they may interact with their targets in a cell-specific manner. S2, derived from embryonic cells of *Drosophila*, do not represent the entire transcriptome of *Drosophila* and may not faithfully recapitulate the cell-specific RBP–RNA interactions. However, they can still be used for studying the mechanistic aspect of these interactions, which are quite well conserved across cells.

3. Users must be aware that the fusion of ADARcd to the RBPs adds approximately 40 KDa to the protein size, which could potentially result in steric hindrance and alter the RNA-binding activity. Experiments may be conducted to determine the optimal location of ADARcd to counter this limitation and improve the efficiency of TRIBE.

4. The critical limitation for RNA-editing-based methods is the presence of endogenous ADAR editing and genomic SNPs. Untransfected samples can be sequenced to subtract the genomic DNA sequence from the same genetic background during the data analysis to account for background noise.

5. As stated earlier, we highly recommend the use of biological replicates to ensure reproducibility.

6. A modified version of TRIBE, called HyperTRIBE, which uses HyperADAR with a mutation at its E488Q position, has been tested and proven to improve the efficiency and specificity of target identification.
